# Molecular Biology: What Is Radiation’s True Target?

**DOI:** 10.1289/ehp.115-a402a

**Published:** 2007-08

**Authors:** Carol Potera

Radiobiologists have long believed that ionizing radiation, like gamma rays, kills cells by shattering DNA. Now Michael Daly, an associate professor of pathology at the Uniformed Services University of the Health Sciences, contends that proteins—not DNA—are the most sensitive targets, at least in some radiation-sensitive bacteria. Moreover, Daly’s team discovered a novel manganese complex that appears to protect proteins from gamma rays. This new perspective comes from working with *Deinococcus radiodurans*, a bacterium nicknamed “Conan the Bacterium” because it survives huge radiation doses.

For 15 years, Daly probed DNA, genes, and chromosomal packaging for clues to how *Deinococcus* defies death. He noticed that *Deinococcus* and other radiation-resistant bacteria accumulated high levels of manganese, compared with radiation-sensitive bacteria. When zapped with the same dose of gamma rays, however, radiation-resistant and radiation-sensitive bacteria all experienced about the same number of double-strand DNA breaks. Daly reported these results in the 5 November 2004 *Science*, then tackled the remaining question: What does manganese protect in radiation-resistant bacteria?

The latest proof-of-concept experiments, published in the April 2007 *PLoS Biology*, showed that manganese neutralized reactive oxygen species (ROS), or free radicals, generated by ionizing radiation. When the researchers bombarded radiation-resistant bacteria with gamma rays, manganese appeared to protect proteins from a form of oxidative damage called carbonylation. But in radiation-sensitive bacteria with little manganese, gamma rays caused high levels of protein oxidation, and the microbes died. Shifting the emphasis from DNA to proteins “is heretical,” Daly admits, “but the data speak for themselves.”

Daly’s surprising results “fly in the face of fifty years of dogma,” says John Battista, a professor of microbiology at Louisiana State University. Gamma rays cause a variety of DNA lesions, and Daly measured only double-strand breaks, one of the least abundant types. “I would be more convinced if he measured a more abundant lesion like thymine glycol levels,” says Battista.

Daly’s laboratory has most recently identified a small manganese complex in *Deinococcus* that is extremely resistant to ionizing radiation and protects proteins but not DNA. Daly plans to pursue practical applications for the manganese complex in mammalian cells, such as protecting people against radiation sickness, or sparing healthy cells from the ravages of radiation during cancer treatment. Many toxicants—including tobacco smoke, ultraviolet light, and heavy metals—damage cells via the production of ROS. If the complex prevents ROS damage to proteins in mammalian cells, too, other applications might someday include a lotion that could protect skin from ultraviolet rays. “All this lies ahead to be proven,” Daly says.

## Figures and Tables

**Figure:**
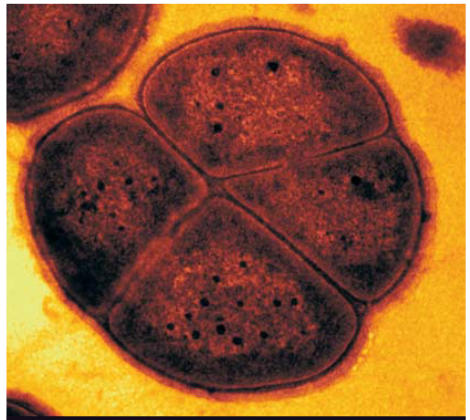
Deinococcus radiodurans

